# Relative Biological Effectiveness of HZE Particles for Chromosomal Exchanges and Other Surrogate Cancer Risk Endpoints

**DOI:** 10.1371/journal.pone.0153998

**Published:** 2016-04-25

**Authors:** Eliedonna Cacao, Megumi Hada, Premkumar B. Saganti, Kerry A. George, Francis A. Cucinotta

**Affiliations:** 1 Department of Health Physics and Diagnostics Sciences, University of Nevada Las Vegas, Las Vegas, Nevada, United States of America; 2 Radiation Institute for Science and Engineering, Prairie View A&M University, Prairie View, Texas, United States of America; 3 Wyle, Houston, Texas, United States of America; Georgetown University, UNITED STATES

## Abstract

The biological effects of high charge and energy (HZE) particle exposures are of interest in space radiation protection of astronauts and cosmonauts, and estimating secondary cancer risks for patients undergoing Hadron therapy for primary cancers. The large number of particles types and energies that makeup primary or secondary radiation in HZE particle exposures precludes tumor induction studies in animal models for all but a few particle types and energies, thus leading to the use of surrogate endpoints to investigate the details of the radiation quality dependence of relative biological effectiveness (RBE) factors. In this report we make detailed RBE predictions of the charge number and energy dependence of RBE’s using a parametric track structure model to represent experimental results for the low dose response for chromosomal exchanges in normal human lymphocyte and fibroblast cells with comparison to published data for neoplastic transformation and gene mutation. RBE’s are evaluated against acute doses of γ-rays for doses near 1 Gy. Models that assume linear or non-targeted effects at low dose are considered. Modest values of RBE (<10) are found for simple exchanges using a linear dose response model, however in the non-targeted effects model for fibroblast cells large RBE values (>10) are predicted at low doses <0.1 Gy. The radiation quality dependence of RBE’s against the effects of acute doses γ-rays found for neoplastic transformation and gene mutation studies are similar to those found for simple exchanges if a linear response is assumed at low HZE particle doses. Comparisons of the resulting model parameters to those used in the NASA radiation quality factor function are discussed.

## Introduction

Estimating high LET radiation carcinogenesis risk is of interest in studies of normal tissue damage in Hadron cancer therapy with protons, carbon and other heavy ion beams, and space radiation protection during space travel. The high charge and energy (HZE) particles of galactic cosmic rays (GCR) include particles from hydrogen to nickel over a broad energy range and through nuclear interactions a significant secondary radiation dose occurs most importantly neutrons [1–3]. Major challenges for high LET risk estimation are the absence of human epidemiology data, and the quantitative and qualitative differences in their biological effects compared to low LET radiation found in experimental studies with murine or cell culture models [1,2]. GCR dose-rates in tissue vary from 0.08 to 0.2 Gy per over the 11-year solar cycle with less than 0.05 Gy/y from HZE particles [1–5]. In Hadron therapy with carbon beams an RBE for cell killing is applied such that the dose per fraction of less than 1 Gy often occurs, while a large range of total doses overall fractions (0 to >10 Gy) occur in normal tissues away from tumor sites [6,7]. Mechanisms of biological damage are likely distinct at high dose compared to low dose, and the smaller signal at low dose is a major obstacle for animal experiments to be performed with statistically significant sample sizes.

Very few animal studies of dose responses for tumor induction from HZE particles have been reported [8–14]. These studies have been limited by the number of particles and energies used, while most studies have been carried out at medium to high doses (>0.1 Gy). Chromosomal aberrations (CA), including simple and complex exchanges [15–20], gene mutation [21–24] and neoplastic transformation [25] have been used as surrogate endpoints to investigate radiation quality effects related to cancer risk estimation. Previously we have shown that human peripheral blood lymphocyte (PBL) cells follow a linear dose response for simple exchanges following HZE particle irradiation for doses as low as 0.01 Gy, which corresponds to less than 1 in 5 particle traversal per cell for the ^16^O, ^28^Si, and ^56^Fe particles considered [26]. CA in lymphocytes cells showed radiation quality dependence that deviated from a simple dependence on LET [18] consistent with track structure models of other endpoints (reviewed in [27]), which suggest that biological effects depend on particle charge and kinetic energy and not LET alone. In contrast to lymphocyte cells, normal human fibroblast cells have a low dose response for HZE particles that was best fit with a supra-linear dose response model, suggesting that non-targeted effect (NTE) mechanisms are at play. NTEs are important at doses corresponding to less than 1 particle traversal per cell (<0.2 Gy), with a linear response accurate at higher doses (0.2 to 1 Gy) [26]. Because fundamental to radiation protection is the assumption of a linear dose response, including defining quality factors based on data for relative biological effectiveness (RBE) factors [15,16], the non-linear dose response has important implications at the doses most likely to occur in space radiation exposures and the doses in normal tissues at risk for secondary cancers in Hadron therapy.

Traditionally, radiation protection has used radiation quality factors or radiation weighting factors based on subjective assessments of the experimental quantity RBE_max_, which compares low dose particle exposures to low dose and dose-rate γ-rays [15,16] in model biological systems. The large uncertainties in RBE_max_ observed in various experiments are due in large part to the ineffectiveness of low dose and dose-rate γ–rays, and hence the large uncertainties in determining their biological effects [27–32]. Therefore for RBE assessments and defining the radiation quality factor function to be used in radiation protection, the NASA approach considers acute γ-ray dose response fit to a linear response at doses near 1-Gy as the reference radiation [28,29]. In this report we analyze CA data in human fibroblast and lymphocyte cells using a parametric track structure model that follows the functional form used in the NASA radiation quality factor function [27–29]. The radiation quality dependence of RBEs for chromosomal aberrations is analyzed and compared to results for RBEs for studies of neoplastic transformation and gene mutation.

## Methods

All experimental data for chromosome aberrations were previously published with the exception of data for ^48^Ti particles in the hTERT immortalized 82–6 human normal skin fibroblasts cells described previously [26], which are reported here (S1 Table). These data are collected in identical fashion to our previous report [26] using the premature chromosome condensation technique to collect chromosomes in the G2/M phase of the cell cycle. In these experiments using chromosome painting of chromosomes 1, 2 and 4, the frequency of aberrations in the painted chromosomes was evaluated as the ratio between aberrations scored and total cells analyzed with standard errors calculated assuming Poisson statistics [19,26]. Simple exchanges include translocations and dicentrics, while complex exchanges were scored as exchanges involving a minimum of three breaks in two or more chromosomes [19,26].

The area of the cell nucleus for human fibroblast and lymphocyte cells were determined to be 162 μm^2^ and 30 μm^2^, respectively [26]. Using the relationship between LET (L, in keV/μm), absorbed dose (D, in Gy) and fluence (F, in μm^2^) while ignoring the contributions from delta-rays in cells not traversed by particles [33], the mean number of particle hits per cell nucleus (H) is given by:

$$F\;=\;\frac{6.24\;D}{L}$$ (1)

$$H\;=\;\frac{6.24\;DA}{L}\;=\;F\;A$$ (2)

Using a generalized linear model, the number of exchanges per cell (Y) was fit to linear and non-targeted effect (NTE) models, as described by the following equations:

$$\begin{array}{cc} Y\;=\;Y_{0}\;+\;\sigma F & \text{Linear model} \end{array}$$ (3)

$$\begin{array}{cc} Y\;=\;Y_{0}\;+\;\sigma F\;+\;\eta I & \text{NTE1 model} \end{array}$$ (4)

$$\begin{array}{cc} Y\;=\;Y_{0}\;+\;\sigma F\;(1-e^{-H})+\;\eta \;e^{-H}I & \text{NTE2 model} \end{array}$$ (5)

where Y_0_ is the estimated number of simple or complex exchanges per cell at a dose of 0 Gy, σ is the biological action cross section as described below, I is the indicator function that irradiation occurred, and η represents the non-targeted effects coefficient, which is parameterized as a function of LET by:

$$\eta \;=\;\eta_{0}\;Le^{-\eta_{1}\;L}$$ (6)

The NTE2 model further takes into account that as dose is increased, fewer cells are bystanders and more cells have particle tracks. For the 82–6 fibroblast cells, both linear and NTE models were evaluated for simple exchanges [26]. On the contrary, only linear dose response models for simple and complex exchange aberrations were assessed for human lymphocyte cells based on the finding of a lack of a deviation from a linear response at low doses in previous analysis [26]. The data sets for HZE particles considered doses predominantly below 1 Gy [18,19,26] thus minimizing possible high dose effects that can lead to downward or upward curvature in dose responses.

In the parametric track structure model [27–29,34] the biological action cross section is given by:

$$\begin{array}{cccc} & \sigma & = & \sigma_{0}P+\frac{\alpha_{\gamma }\;L}{6.24} \end{array}(1-P)\begin{array}{ccc} & P= & (1-\text{exp}(-\frac{Z^{*2}}{\kappa \beta^{2}} \end{array}){)}^{m}$$ (7)

where Z* is the effective charge number of the particle, and β is the particle velocity relative to the velocity of light. The constant α_γ_ is the linear regression coefficient for acute doses of γ-rays for the same endpoint. The parametric form of (eq 7) is similar to the Katz cellular track structure model [35,36], however assuming an initial linear dose component for γ-rays for m>1.

The value of m represents the so-called target number which can be fit to the γ-ray dose response data along with a radio-sensitivity parameter D_0_ using

$$\begin{array}{cccc} & Y & = & Y_{0}+{\left[1-\text{exp}(-D/D_{o})\right]}^{m} \end{array}$$ (8)

For RBE estimates against acute doses of γ-rays as the reference radiation, we consider α_γ_ the low-LET slope estimated by assuming a linear dose response at higher doses. This approach is used in the NASA QF model in order to be consistent with the linear response model used to represent the Atomic-bomb survivor solid cancer incidence data [37,38], while in this report we follow a similar approach based on the experiments under consideration.

The value of Y_0_, track structure model parameters (σ_0_ and κ) and NTE model parameters (η_0_ and η_1_) are fitted across all particle beams using nonlinear least-squares data fitting weighted by the inverse of the variance. Using the same approach the values of *m* and *D*_*0*_ or alternatively *α*_*γ*_, are estimated using the data for γ-rays. All statistical analysis and data fitting were done using STATA/SE version 14.1 (Stata Corp.). The best fit for different models was determined using the Akaike information criteria (AIC) and Bayesian information criteria (BIC), which considers the number of parameters in each model, while the lowest AIC and BIC provides the best fit to data [39,40].

The functional form of the RBE function based on the linear or targeted effects assumption (TE), which uses a linear fit to acute γ-ray responses as the reference radiation is [27,41]:

$$\begin{array}{cccc} & RBE_{TE} & = & \begin{array}{ccc} (1-P)+\frac{6.24\sigma_{0}P}{\alpha_{\gamma }L} & & \end{array} \end{array}$$ (9)

For the NTE1 model it is convenient to define a cross-over dose where the contributions from the TE and NTE terms in (eq 4) are equal:

$$\begin{array}{cccc} & D_{cr} & = & \frac{\eta L}{6.24\sigma } \end{array}$$ (10)

The RBE in the NTE1 model is then a function of the particles absorbed dose, D and is given by [27,41]:

$$\begin{array}{ccc} & RBE_{NTE} & =\begin{array}{cc} RBE_{TE}(1+\frac{D_{cr}}{D}) & \end{array} \end{array}$$ (11)

A similar expression is found for the NTE2 model with the modifications for particle hits from (eq 5). At significantly low particle doses (<1 mGy) the NTE contribution should become negligible, and a further low dose modification would be needed as discussed below.

## Results

We first consider the results for human lymphocyte cells exposed to γ-rays. Table 1 show the results of fitting γ-ray dose response data for the same volunteer used in the majority of HZE particle experiments for fixed values of *m* or allowing the value of *m* to be used as a free parameter. Prior results did not reveal a significant difference in the linear slopes for exchanges for the small number of volunteers used in these experiments [18,19,26]. Interestingly values of *m* = 2 and 4 for simple and complex exchanges, respectively, were found to give optimal fits and close to the value found with *m* treated as a free parameter (1.89±0.64 and 3.8±1.3 for simple and complex exchanges, respectively). These values would be suggested if the target number corresponded to the number of double-strand breaks (DSBs) required for each type of exchange. The values of *m* = 2 and 4 for simple and complex exchanges, respectively were used in the analysis of the HZE particle data and RBE predictions.

**Table 1:** Parameter estimates for γ-ray dose response data for simple and complex exchanges in human lymphocytes cells. Parameter and standard deviations (including p-values) for the same volunteer used in the majority of particle experiments are shown. Fits for fixed values of *m* or allowing the value of *m* to be used as a fitting parameter are described. Also shown are values for the Akaike information criteria statistic (AIC) and Bayes information criteria (BIC) statistic where models with the lowest AIC or BIC values provide the optimal fit to the data. The model providing the best fit to experimental data is shown in bold-face font.

	Best fit with “m” free parameter	m = 2	m = 3	m = 4	m = 5
	**Simple Exchanges in Human Lymphocyte Cells**				
**m**	1.89±0.64 (<0.060)	**2**	3	4	5
**D**_**0**_**, Gy**	1.96±0.92 (<0.121)	**1.82±0.17(<10**^**−4**^**)**	1.18±0.11(<10^−4^)	0.92±0.9(<10^−4^)	-
**Adj. R**^**2**^	0.99998	**0.99998**	0.99997	0.99996	
**AIC**	-21.22	**-21.22**	-19.53	-17.8	-
**BIC**	-21.61	**-21.61**	-19.92	-18.2	-
	**Complex Exchanges in Human Lymphocyte Cells**				
**m**	3.8±1.3(<0.063)	2	3	**4**	5
**D**_**0**_**, Gy**	2.33+0.97(<0.092)	6.67±1.33(<0.004)	3.13±0.29(<0.001)	**2.22±0.2(<10**^**−4**^**)**	1.79±0.16(<10^−4^)
**Adj. R**^**2**^	0.99999	0.99999	0.99999	**0.99999**	0.99999
**AIC**	-34.9	-34.45	-36.38	**-36.88**	-36.22
**BIC**	-35.68	-34.84	-36.77	**-37.27**	-36.61
**Simple Exchanges in 82–6 Fibroblasts**					
**m**	**1.18±0.19(<0.025)**	2	3	4	5
**D**_**0**_**, Gy**	**2.67±0.44(<0.026)**	1.66±0.14(<0.001)	1.18±0.26(<0.005)	0.91±0.16(<0.011)	**-**
**Adj. R**^**2**^	**0.9397**	0.7944	0.6205	0.5327	-
**AIC**	**-44.28**	-38.12	-35.06	-34.02	-
**BIC**	**-45.46**	-38.9	-35.84	-34.8	
**HPRT Mutations in V79 Cells**					
**m**	1.35±0.22(<0.122)	**2**	3	4	5
**D**_**0**_**, Gy**	10938±13126(<0.577)	**848.4±44.0(<10**^**−4**^**)**	153.1±8.97(<10^−4^)	64.7±3.77(<10^−4^)	-
**Adj R**^**2**^	0.9324	**0.8694**	0.6722	0.5023	
**AIC**	-154.82	**-150.93**	-144.49	-141.57	-
**BIC**	-154.92	**-150.99**	-144.54	-141.62	-

A linear dose response model fit to γ-ray data led to the coefficients, α_γ_ of 0.157±0.027 Gy^-1^ and 0.015±0.006 Gy^-1^ for simple and complex exchanges, respectively in PBL cells.

**Fig 1:**
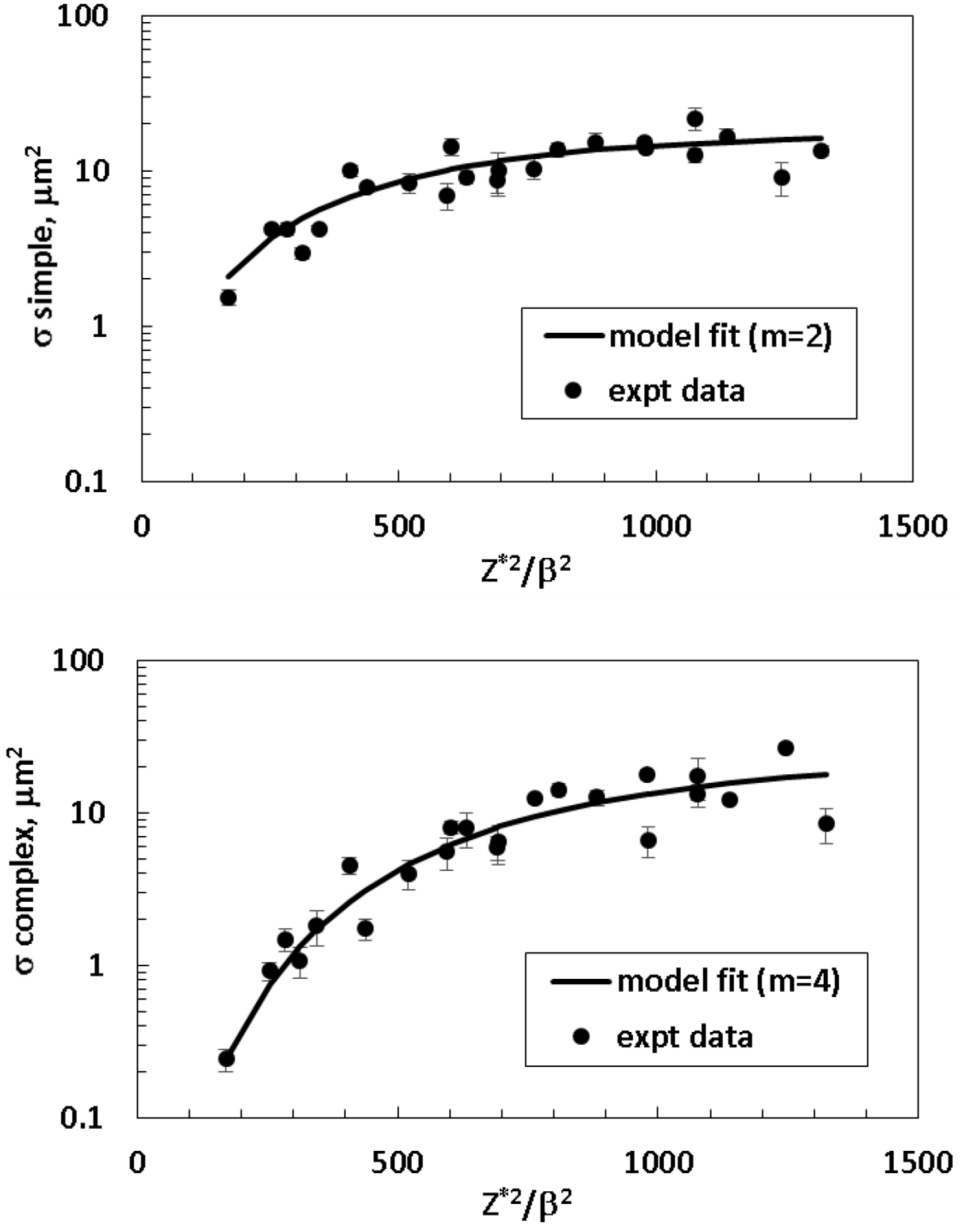
Action cross sections for simple exchanges (upper panel) and complex exchanges (lower panel) versus Z*^2^/β^2^ for human peripheral blood lymphocytes (PBL).

The biological action cross section represents the probability of damage induction per particle. Fig 1 shows the model fits to the biological action cross section for simple (upper panel) and complex exchanges (lower panel), respectively in PBL cells. Experimental data considered included those for ^16^O (55, 77, 128, and 250 MeV/u), ^20^Ne (64, 89, 142, and 267 MeV/u), ^28^Si (93, 150, 170, 240, 490, and 600 MeV/u), ^48^Ti (240, 380, and 1000 MeV/u), and ^56^Fe (450, 600, 750, 1000, and 5000 MeV/u) particles. In Table 2 we show the values of parameters resulting from these fits. The value of σ_0_ which roughly corresponds to the geometric cross sectional area where damage occurs or saturation value of the cross section, was found as 17.7 and 22.9 μm^2^ for simple and complex exchanges, respectively. These values represent a significant fraction of the nuclear area of the lymphocyte cells of ~30 μm^2^, with the value for complex exchanges somewhat larger than that found for simple exchanges.

**Table 2:** Parameter estimates and standard deviations for several models fit to experimental data for human lymphocyte cells with p-values for significance shown in parenthesis.

Parameter	Simple Exchanges	Complex Exchanges
**Y**_**0**_	0	0
**m**	2	4
**σ**_**0**_**, μm**^**2**^	17.7±2.7 (<10^−4^)	22.9±4.55 (<10^−4^)
**κ**	445.9±62.4 (<10^−4^)	478.2±57.4 (<10^−4^)
**Adjusted R**^**2**^	0.9976	0.9962

**Fig 2:**
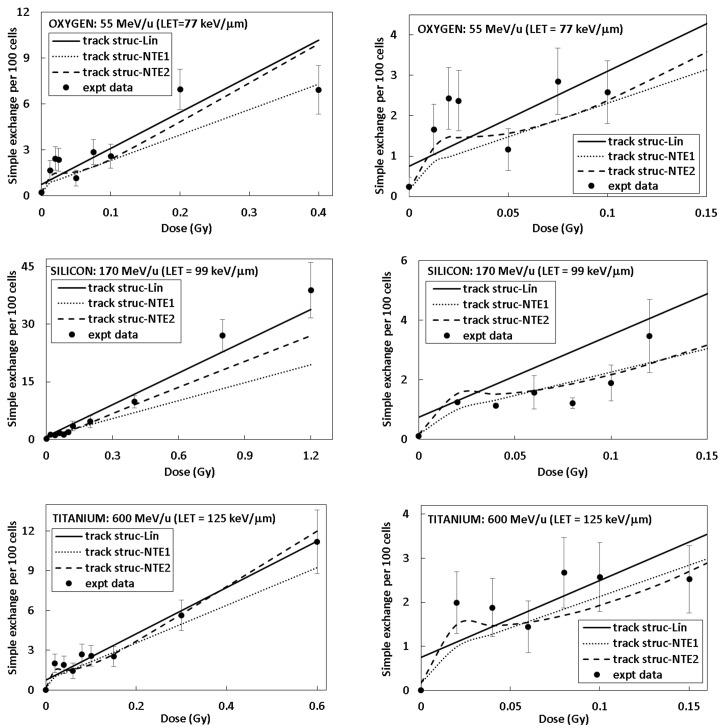
Frequency of simple exchanges per 100 cells as a function of dose for 82–6 human fibroblast cells. Experimental data shown with symbols and model fits by lines. (Panel A: O (55 MeV/u) results over all doses, panel B: O (55 MeV/u) results at lower doses, panel C: Si (170 MeV/u) results over all doses, and panel D: Si (170 MeV/u) results at lower doses), and panel E: Ti (600 MeV/u) results over all doses, and panel F: Ti (600 MeV/u) results at lower doses).

**Fig 3:**
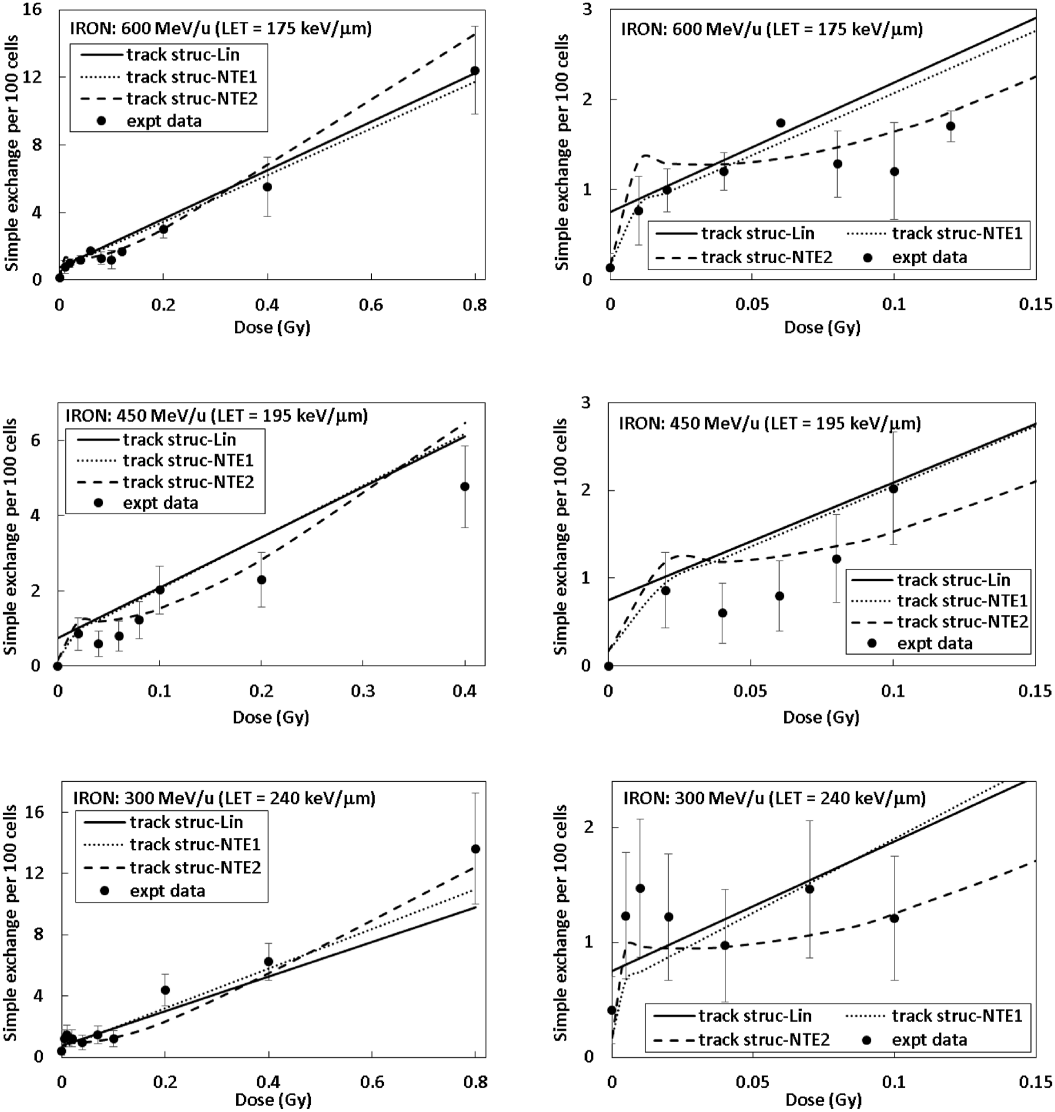
Frequency of simple exchanges per 100 cells as a function of dose for 82–6 human fibroblast cells. Experimental data shown with symbols and model fits by lines. Panel A: Fe (600 MeV/u) results over all doses, panel B: Fe (600 MeV/u) at lower doses, panel C: Fe (450 MeV/u) results over all doses, panel D: Fe (450 MeV/u) results at lower doses, panel E: Fe (300 MeV/u) results over all doses, and panel F: Fe (300 MeV/u) results at lower doses.

Figs 2 and 3 show results of fitting linear and NTE’s dose response models to experimental data for simple exchanges in 82–6 fibroblast cells for several HZE particle types and energies. The left panels of Figs 2 and 3 show the full dose range and the right panels focus on the lower dose range up to 0.15 Gy. Results of fitting parametric models to the γ-ray data for simple exchanges for 82–6 human fibroblast cells are also shown Table 1. The value of *m* = 2 provided an adequate fit however a value of 1.2 yielded a slightly better fitting value, which indicates a significant linear dose component. The α_γ_ value for a linear fit was found as 0.041±0.0051 Gy^-1^. Table 3 shows results for our comparison of the linear and NTE models to the HZE particle data. The NTE2 model provided the best fit based on the AIC and BIC criteria. In Table 3 a common Y_0_ parameter is assumed for all particle beams in fits of each of the different models. The value of σ_0_ of 6.75 μm^2^ for the NTE2 is much smaller than found for simple exchanges in lymphocyte cells (17.7 μm^2^), which likely reflects the geometric differences between the flat ellipsoidal and spherical shapes of fibroblasts and lymphocytes, respectively, or some underlying difference in radiosensitivity including differences in recombination events leading to exchanges for these two cell types.

**Table 3:** Parameter estimates and standard deviations for several models fit to experimental data for 82–6 human fibroblast cells in linear and non-targeted (NTE) models. Shown are Akaike information criteria statistic (AIC) and Bayes information criteria (BIC) statistic values for the different models are for global fit for all particle data in this cell line with values in bold font providing best fit to data. P-values that indicate the significance of the parameters are shown in parenthesis.

Parameter/Model	Linear	NTE1 Model	NTE2 Model
Y_0_	0.0075 +0.0008	0.0017±0.0007	0.0017±0.0007
m	2	2	2
σ_0_, μm^2^	4.44+1.17 (<0.019)	6.12 ±1.66 (<0.021)	6.75±1.67 (<0.016)
κ	392±255 (<0.001)	796±287 (<10^−4^)	590±236 (<10^−4^)
η_0,_ (keV/μm)^-1^	-	0.00011±0.00009(<0.299)	0.00047±0.00026(<0.152)
η_1_, (keV/μm)^-1^	-	0.007±0.0056(<0.256)	0.011±0.0035(<0.036)
AIC	3.29	3.24	**3.17**
BIC	-12.20	-13.50	**− 13.59**

**Fig 4:**
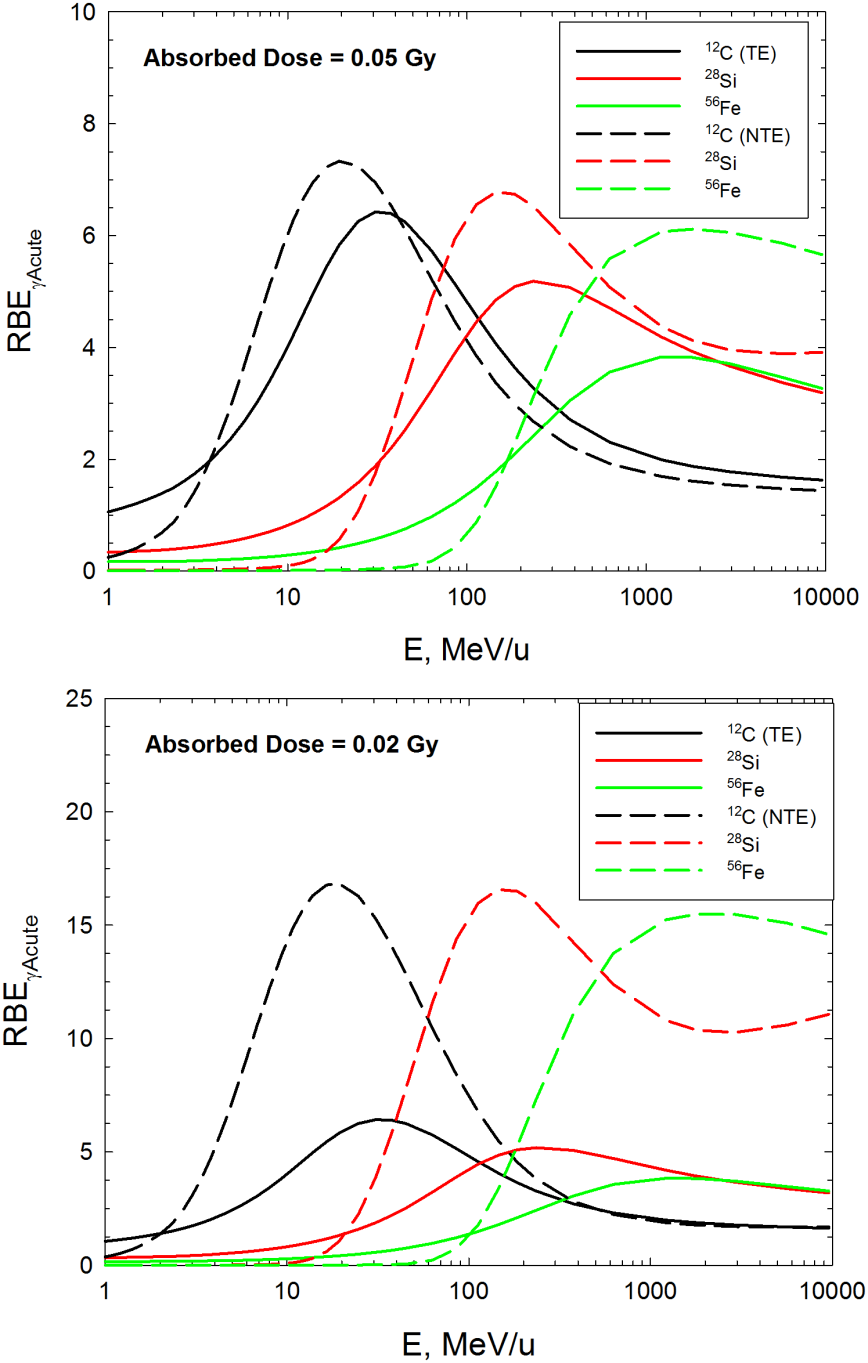
Predictions of relative biological effectiveness, RBE_γAcute_ for simple exchanges in 82–6 fibroblasts in targeted effects (TE) model and non-targeted effects (NTE2) models. A) Upper panel is predictions for absorbed dose of 0.05 Gy for C, Si, and Fe particles. B) Lower panel is predictions for absorbed dose of 0.02 Gy for C, Si, and Fe particles.

**Fig 5:**
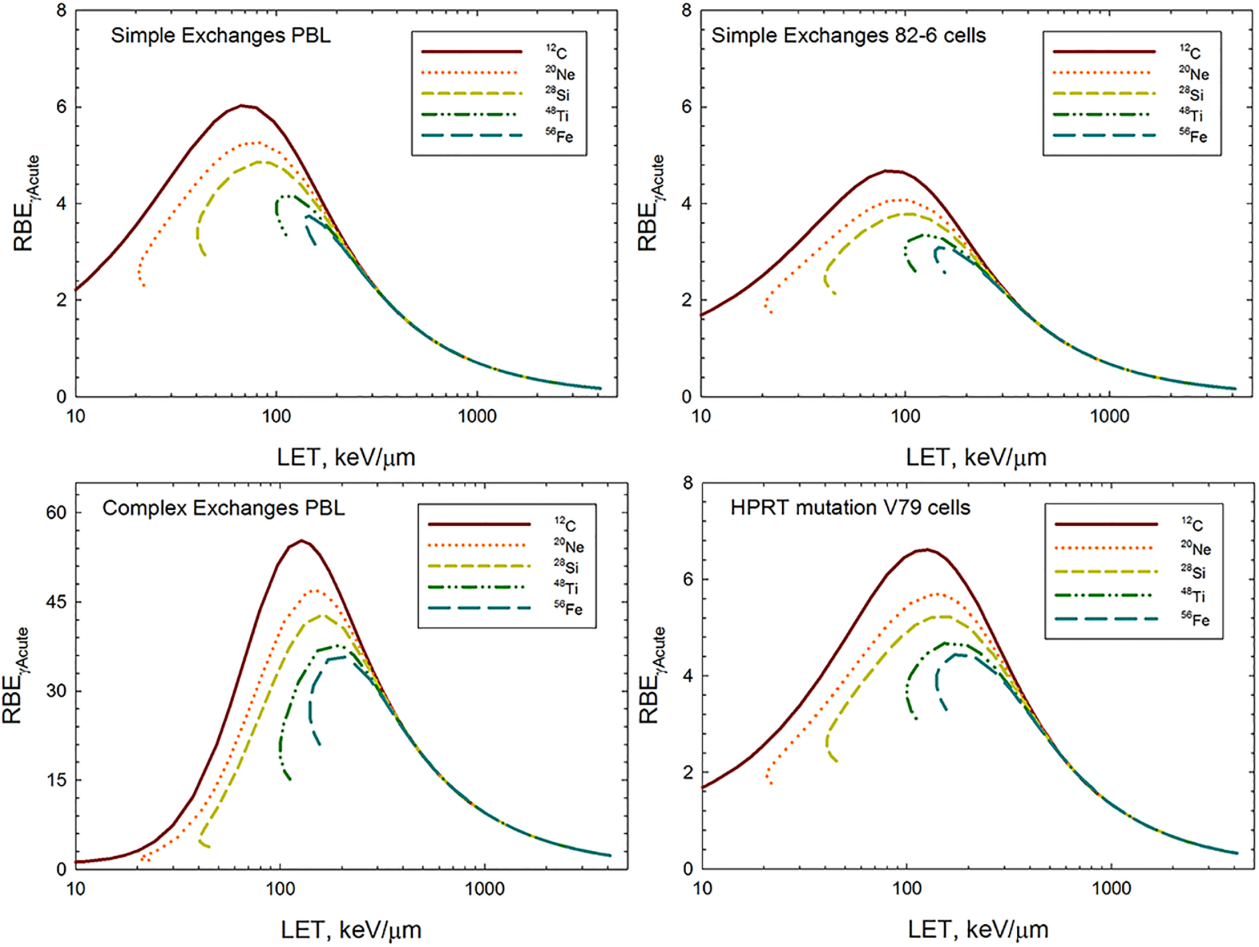
Predictions of relative biological effectiveness, RBE_γAcute_ versus LET in targeted effects (TE) model. Results showing branching of LET with charge number for C, Ne, Si, Ti and Fe particles are shown for A) Simple exchanges in lymphocytes, B) simple exchanges in 82–6 fibroblast cells, C) complex exchanges in lymphocyte cells, and D) HPRT mutations in V79 cells.

Using the model equations and fitted parameter values, we are able to make predictions of RBE for all particle types versus kinetic energy or LET where we assume a linear fit to the effects of acute doses of γ-rays as the reference radiation, denoted as RBE_γAcute_. Fig 4 shows predictions for simple exchanges in 82–6 cells in the TE model and the NTE model for absorbed dose of 0.05 Gy (upper panel) and 0.02 Gy (lower panel), where RBE’s are predicted to be much higher compared to the TE model. In the NTE model the RBE values are dose-dependent down to a very low dose and limit to the TE estimate of RBE at higher doses (>0.2 Gy). A similar result was found for RBE’s for Harderian gland tumors [13,27,41]. Fig 5 shows predictions of RBE_γAcute_ for simple exchanges in lymphocytes (Panel A) and 82–6 fibroblasts ignoring the NTE contribution (Panel B) and complex exchanges in lymphocytes (Panel C). The RBE_γAcute_ values are much lower than our previous reports estimating RBE_max_ using low dose or dose-rate γ–ray responses as the reference radiation [18,19]. Large RBE values (>30) for complex exchanges in lymphocytes occur due to the ineffectiveness of acute doses of γ-rays. Interestingly complex exchanges in 82–6 fibroblasts were rare events at low particle doses (<0.2 Gy) thus precluding dose response and RBE modeling.

Experiments investigating heavy ion induction of HPRT gene mutation in V79 Chinese hamster cells by several groups [21–24] considered a large number of particle types and energies, which are compared to our results for chromosomal exchanges. We fit the TE model to data for biological action cross section for particles with charge number from Z = 1 to 28, while ignoring experiments with Z>28, which provides data for 45 particle type and energy combinations. Table 1 also shows the fits to the γ-ray data from Kiefer et al. [21]. Inter-laboratory differences in the γ-ray response occur and because the current focus is on HZE particles we used the Kiefer *et al*. γ-ray dose response data for RBE estimates. Table 1 shows results with *m* = 2 found as the best fit for integer *m* values. We note that considering *m* as a free parameter results in *m* = 1.35±0.22, however the resulting value of D_0_ (10,938±13,126 Gy, with p-value<0.577) is poorly fit. Based on biophysical consideration this value for D_0_ is too large but at least an order of magnitude when one considers expected microscopic energy deposition and the physical size of the HPRT gene [42], and we therefore consider the *m* = 2 case as the optimal fit. A linear fit to the Kieffer *et al*. γ-ray data for the restricted range of doses of 1 to 4 Gy yielded α_γ_ = (5.61 + 0.788) x 10^−6^ Gy^-1^. For *m* = 2 the value of σ_0_ = (1.2±0.13) x10^-3^ μm^2^ is obtained, with a nearly identical fitted σ_0_ value for *m* = 3 and 4 (results not shown). We found that *m* = 2 provided a slightly improved fit compared to *m* = 3 or 4 for the published action cross section values from [21–24] and this choice is also preferred based on the γ-ray dose response data. Using the *m* = 2 parameter set, Fig 5 (Panel D) shows the resulting predictions of RBE_γAcute_ for HPRT mutations in V79 cells. Comparison of the different predictions in Fig 5 indicates a substantial shift in RBE as a function of LET for the different HZE particles considered.

We next considered a comparison of the values of κ obtained for the different surrogate endpoints in cell culture models. For comparison we also considered published values of fits to the neoplastic transformation experiments for 9 HZE particle type and energy combinations [25] with *m* = 2 and 3 fits made by Waligorski *et al*. [43]. Table 4 shows how values of κ vary with *m* for the different endpoints and the average value of κ for the different surrogate endpoints. The trend of a functional κ(m) relationship is similar to that of the probability distribution function (PDF) representing model parameter uncertainty assumed in the NASA QF model [27] where for m = 2, 3, and 4 subjective estimates of κ = 733, 550, and 440 are assumed, which are reasonably close to the average values obtained here of κ = 630±142, 534±81, and 439±91, respectively. However in the NASA model, the QF function assumed the value of *m* = 3 as the central estimate, while the present analysis suggest *m* = 2 is the best choice based on fits to published HZE particle experiments where a significant number of particle types were available to test model parameters.

**Table 4:** Estimates of parameter κ from (eq 7) for several surrogate endpoints that result after fitting data with fixed values for the target number (*m*). TE is the linear also called targeted effects model of (eq 5) and NTE2 is the non-targeted effects model of (eq 7). The average overall model uses the TE results for simple exchanges in 82–6 fibroblast cells. *Standard deviations (SD) for the values fit to the transformation experiment were not reported by Waligorski *et al*. [43] and we assumed a SD of 35% of the central estimate based on the maximum of results for other endpoints considered.

Experimental Cell/Endpoint	m = 2	m = 3	m = 4
**Simple exchanges, Lymphocytes**	446±62	302±27	245±17
**Complex exchanges, Lymphocytes**	-	773±147	478±57
**Simple exchanges in 82–6 Fibroblast cells, TE model**	444±117	428±96	-
**Simple exchanges, 82–6 Fibroblasts cells, NTE2 model**	590±236	416±129	-
**HPRT mutation, V79 Cells**	880±308	692±215	596±173
C3H10T1/2 neoplastic transformation*	750	475	-
**Average for TE models**	**630±142**	**534±81**	**439±91**

## Discussion

In this report we focused on the radiation quality dependence of RBE’s for surrogate endpoints of cancer risk in cell culture models, and how RBE values are influenced by possible NTEs. Very few animal experiments of the dose response for tumor induction have been reported with particle beams [8–14]. Thus investigations of surrogate endpoints for cancer risk utilizing a sufficient number of particles (>5) play an important role in making an assessment of radiation quality for secondary cancer risk in Hadron therapy and space travel, which should be augmented with theoretical understanding of particle track structure and microscopic energy deposition. Our approach considered the parametric track structure model that is used in the NASA QF function [27–29], which depends on particle kinetic energy and charge number and not LET alone as assumed in previous ICRP and NCRP considerations [2,15,16]. The NASA risk model has recently been updated to use acute doses of γ-rays as the reference radiation [28], which results in a lower overall uncertainty in risk estimates [29]. The RBE’s reported in this report use this same approach.

Not all aspects of particle track structure are represented in this functional form of (eq 7), including the influence of the stochastics of particle tracks [44] and the role of differences in the energies of delta-rays [45] that occur between low and high velocity particles, which are possibly important because electrons are more biologically effective at low (< 10 keV) compared to higher energies [15]. However, the model allows for a dependence on both charge number Z, and kinetic energy, E, which is supported by experiments. Thus the predictions of Fig 5 result in a significant deviation of radiation quality dependence of LET alone. The value of σ_0_ found for each endpoint study reflects the geometric area of the damage region for the endpoint. The value of σ_0_ determined for complex exchanges was larger than for simple exchanges in human lymphocytes, which is reflective of the larger number of DSB’s needed for complex exchange formation. A key aspect of the NASA model is to form probability distribution functions (PDF’s) to represent uncertainties in values of parameters that enter into the model function. The present work contributes to making an objective assessment of PDF’s for NASA QF function parameters as recommended in a review by the U.S. National Research Council [31].

The results of fits for simple and complex CAs in PBL were accurately represented by target numbers of *m* = 2 and *m* = 4, which suggests mis-repair of 2 and 4 double strand breaks, respectively are the dominant cause of these endpoints. Results for simple and complex chromosomal exchanges in PBL and fibroblast cells were compared to results of published studies for HPRT mutations and neoplastic transformation were a significant number (45 and 9, respectively) of HZE particle species and energies were considered. This comparison indicated that the values of κ parameter in the NASA QF function model is consistent with the average of the surrogate endpoint data considered, however the comparison also suggests the value of *m* = 2 is more accurate than the central estimate of *m* = 3 used in the NASA QF function. Thus the present analysis when combined with results for tumor induction after high LET radiation can be used to update the QF function model parameters and their associated PDF’s of uncertainty.

In future work new experimental data on Harderian gland tumorigenesis [13] will be utilized to further consider these values. The outcome of these differences in QF function parameters can modify predictions of space radiation risks and shielding effectiveness evaluations, which depend critically on the QF function and its inherent uncertainties. Other parameters values corresponding to the maximum values of the QF function and the dose and dose-rate reduction effectiveness factor (DDREF) have been considered in recent reports based on published mouse tumor induction studies with γ-rays, particles and fission neutrons [27,28,46]. Our use of RBE_γAcute_ instead of RBE_max_ is consistent with approaches to model human epidemiology data [37,38], while reducing uncertainties in model parameters and avoiding controversies in estimating whether or not low dose and dose-rate γ-rays have a linear dose component.

The second focus of this report is to consider the relative contribution of TE and NTE’s to simple exchanges in the 82–6 fibroblast cells and the resulting influence of NTEs on RBE predictions. Studies of NTEs including bystander effects and genomic instability in the progeny of irradiated cells [47–55] have challenged the traditional radiation protection paradigm of dose responses that increase linearly with dose, which are motivated by a DNA damage mutational mechanism or other targeted DNA effects. Bystander experiments for micronuclei, sister chromatid exchanges, neoplastic transformation and genomic instability *in vitro* suggests that NTE may occur with a shallow or nearly constant dose response above a very low dose threshold (<0.01 Gy) [53]. Experimental results at low doses (<0.1 Gy) of high LET radiation are sparse hindering the assessment of the relative contributions of TE and NTE to radiation cancer risk at low dose or for chronic radiation exposures. A linear dose response model can be argued on the basis of DNA damage and mutation concepts; however the experimental basis for a linear response model is not strong with very few experiments performed with multiple low doses defined as less than one particle per cell nucleus. Most studies have used higher doses or at most a single low dose, which precludes understanding the role of NTEs.

Our results (Fig 5) show that RBE’s may be significantly under-estimated at low doses if NTEs occur. RBE’s at doses of 0.05 and 0.02 Gy were chosen for comparison as these doses occur near the transition point where TEs (>0.05 Gy) and NTEs (<0.05 Gy) are predicted to dominate. At higher doses (>0.05 Gy) the results for the TE and NTE models become similar and independent of dose. A similar result is found for new studies of Harderian gland tumors induced by low doses of particle irradiation [13]. At sufficiently low doses we expect NTEs to diminish to zero. Prior studies suggest this would occur at absorbed doses of about 1 mGy or lower [47–55]. Further study is needed in this area to understand how RBEs should be modeled at very low doses and especially for modeling chronic radiation exposures.

The published data for HPRT mutations or neoplastic transformation [21–25] did not consider a sufficient number of low doses to make an assessment of possible NTEs. However studies of HPRT mutations following α-particle irradiation found a significant bystander effect, while mutations in directly irradiated cell show predominantly large scale deletions and those found in bystander cells reveal primarily point mutations [47]. A more recent study by Liber et al. [55] of TK mutations in WTK1 lymphoblastoid cells exposed to several HZE particle types showed bystander effects using the medium transfer method. Similarly an increased frequency of spontaneous neoplastic transformation in the progeny of bystander cells has been reported following ^56^Fe particle irradiation of C3H10T1/2 cell cultures [56].

In conclusion, we have considered several experimental data sets for surrogate endpoints of cancer risk, including chromosomal aberrations, gene mutation and neoplastic transformation, focusing on experiments where a significant number (>5) of HZE particle dose responses were reported thus allowing for model fits of the radiation quality dependence of RBEs. Taken together our analysis of these data sets provides detailed information on how cancer initiation events such as large scale genomic rearrangements and deletions depend on radiation quality and dose. Unfortunately there is little data related to cancer promotion and progression and their dependences on radiation quality available for analysis. At this time, studies are inconclusive on whether or not NTE’s will influence RBEs for tumor induction because of insufficient experimental studies at low dose (<0.1 Gy). However this topic is very critical for risk assessment in Hadron therapy and space travel. In future work the approach developed herein will be integrated with evaluations of more limited data for mouse tumor induction, including exploring the application of alternate models of particle track structure to represent radiation quality effects.

## Supporting Information

S1 Table Whole genome equivalents of the frequency of chromosomal aberrations. Shown are the number of cells scored, means and standard errors for simple complex, and total exchanges per 100 cells in 82–6 human fibroblast cells for ^48^Ti particles (Energy of 600 MeV/u; LET of 125 keV/μm)
(DOCX)
