# Supplementary material for: Relative Biological Effectiveness of HZE Particles for Chromosomal Exchanges and Other Surrogate Cancer Risk Endpoints
Source: PLoS One. 2016 Apr 25;11(4):e0153998. doi: 10.1371/journal.pone.0153998 (PMC4844187; doi:10.1371/journal.pone.0153998)
Supplement: S1 Table — Shown are the number of cells scored, means and standard errors for simple complex, and total exchanges per 100 cells in 82–6 human fibroblast cells for 48Ti particles (Energy of 600 MeV/u; LET of 125 keV/μm) (DOCX) [file pone.0153998.s001.docx]

**Supplementary Table S1.** Whole genome equivalents of the frequency of chromosomal aberrations. Shown are the number of cells scored, means and standard errors for simple complex, and total exchanges per 100 cells in 82-6 human fibroblast cells for ^48^Ti particles (Energy of 600 MeV/u; LET of 125 keV/μm).

| Dose (Gy) | Cells scored | Frequency of chromosome aberrations | | |
| --- | --- | --- | --- | --- |
|  |  | Simple | Complex | Total |
| 0 | 1008 | 0 | 0 | 0 |
| 0.02 | 998 | 1.99 ± 0.70 | 0 | 2.23 ± 0.74 |
| 0.04 | 1055 | 1.88 ± 0.66 | 0.23 ± 0.23 | 2.11 ± 0.70 |
| 0.06 | 1034 | 1.44 ± 0.59 | 0.24 ± 0.24 | 1.68 ± 0.63 |
| 0.08 | 1022 | 2.67 ± 0.80 | 0 | 2.67 ± 0.80 |
| 0.10 | 1060 | 2.57 ± 0.78 | 0.23 ± 0.23 | 2.81 ± 0.81 |
| 0.15 | 1041 | 0.95 ± 0.48 | 0 | 0.95 ± 0.48 |
| 0.30 | 1055 | 5.64 ± 1.15 | 0.23 ± 0.23 | 5.87 ± 1.17 |
| 0.60 | 487 | 11.19 ± 2.39 | 0.51 ± 0.51 | 11.70 ± 2.44 |
